# Gelseminum

**Published:** 1874-08

**Authors:** M. W. Morton

**Affiliations:** Vernon, Alabama


### GELSEMINUM.

*Editors of Record:*

Permit me, through your journal, to say something relative to the above article. The reason for so doing, is from the simple fact that quite a number have been writing on the article that scarcely know anything about it, and cannot lay down the indication for its use. Gelseminum, when indicated, is one of the most valuable remedies in use, and when not indicated, is an unsafe article. I have used it for quite a number of years, and invariably prepare my own tincture; and the best time to collect the root is April and May. So soon as gathered and washed should be bruised, and for every viij. oz. of the root, add oj. of alcohol, 70 per cent., and set aside for two or three weeks, after which pour off and filter. This forms a tincture of a beautiful violet-tint; it has a peculiar odor, somewhat resembling that of new honey, and a faint, peculiar, not unpleasant taste.

The indications for the use of tincture gelseminum: *Flushed face, bright eyes, contracted pupils.* Where ever you find the above, it does not matter what you may call the disease, it is *the* remedy. It is the remedy in determination of the blood to the brain; quiets irritation and gives rest; it lessens the frequency of the heart's action, and removes obstruction to the free flow of blood; hence is a sedative. It also increases the secretions in same way. It is contra-indicated where the circulation is feeble and there is tendency to congestion. Never give it if the eyes are dull, pupils dilated, and the countenance expressionless.

The effects of an overdose, or where the system is thoroughly under the influence, is dullness about keeping the eyes open; double vision and blindness, sometimes giving rise to difficulty in deglutition. These symptoms will pass off very readily by the inhalation of ammonia, and stimulants internally.

Some persons will feel the effects of very small doses, whilst others can take with impunity much larger. Hence, the best plan of giving it, is in small doses, often repeated; increase or diminish, according to effect. I generally prescribe for an adult gtta. x to xx, every one and a half or two hours; children, gtta. iij. to v. There is no danger in *gelseminum*, when the indications are as set forth above. A majority of the *cases will need no other remedy.*

M. W. Morton, M.D,

Vernon, Alabama.

